# Current advances in 2025: A critical review of selected topics by the Association for the Advancement of Blood and Biotherapies (AABB) Clinical Transfusion Medicine Committee

**DOI:** 10.1111/trf.70381

**Published:** 2026-08-31

**Authors:** Nabiha H. Saifee, Jennifer Andrews, Mary Berg, Stella T. Chou, Mischa Covington, Elizabeth P. Crowe, Ruchika Goel, Juliana A. Guarente, Tina Ipe, Jeremy W. Jacobs, Jessica Jacobson, Cyril Jacquot, Jenna Khan, Thane A. Kubik, Theodros Mamo, Michael F. Murphy, Kerry O'Brien, Monica B. Pagano, Anil K. Panigrahi, Jacqueline N. Poston, Scott E. Potter, Simon J. Stanworth, Moritz Stolla, Alyssa Ziman, Ryan A. Metcalf

**Affiliations:** ^1^ Department of Laboratory Medicine and Pathology Seattle Children's and University of Washington Seattle WA USA; ^2^ Department of Pathology, Microbiology, and Immunology Vanderbilt University Nashville Tennessee USA; ^3^ Department of Pediatrics Vanderbilt University Nashville Tennessee USA; ^4^ Department of Pathology University of Colorado School of Medicine and UCHealth Aurora Colorado USA; ^5^ Department of Pediatrics University of Pennsylvania Philadelphia Pennsylvania USA; ^6^ Department of Pathology Brigham and Women's Hospital and Harvard Medical School Boston Massachusetts USA; ^7^ Department of Pathology Johns Hopkins University Baltimore Maryland USA; ^8^ Carter BloodCare Bedford Texas USA; ^9^ Department of Pathology and Genomic Medicine Thomas Jefferson University Hospital Philadelphia Pennsylvania USA; ^10^ Department of Pathology University of Arkansas Little Rock Arkansas USA; ^11^ Department of Pathology NYU Grossman School of Medicine New York New York USA; ^12^ Children's National Hospital Washington DC USA; ^13^ Department of Pathology University of Virginia Charlottesville Virginia USA; ^14^ Department of Pathology and Laboratory Medicine University of Calgary Calgary Alberta Canada; ^15^ OhioHealth Arthur G.H. Bing, MD, Cancer Center Blood and Marrow Transplant and Cellular Therapy Program Columbus Ohio USA; ^16^ NHS Blood and Transplant, Oxford University Hospitals NHS Foundation Trust, and the University of Oxford Oxford UK; ^17^ Department of Pathology Beth Israel Deaconess Medical Center and Harvard Medical School Boston Massachusetts USA; ^18^ Department of Laboratory Medicine and Pathology University of Washington Seattle WA USA; ^19^ Department of Anesthesiology, Perioperative and Pain Medicine, and Pathology Stanford University School of Medicine Stanford California USA; ^20^ Department of Pathology and Laboratory Medicine University of Vermont Burlington Vermont USA; ^21^ Department of Medicine University of Vermont Burlington Vermont USA; ^22^ Department of Pathology and Laboratory Medicine Geisel School of Medicine at Dartmouth Lebanon New Hampshire USA; ^23^ Bloodworks Northwest Research Institute Seattle WA USA; ^24^ Department of Pathology and Laboratory Medicine David Geffen School of Medicine at UCLA Los Angeles California USA; ^25^ Department of Pathology University of Utah Salt Lake City Utah USA

**Keywords:** apheresis, biotherapies, clinical transfusion medicine, health care disparities, hemoglobinopathies, hemostasis, immunohematology, genomics, infectious diseases, patient blood management, pediatric transfusion medicine, platelets, red blood cells, transfusion

**Abbreviations**
AABB: Association for the Advancement of Blood and Biotherapies
AMI: acute myocardial infarction
BLC: Biotherapies Leadership Committee
CTMC: Clinical Transfusion Medicine Committee
CPGs: clinical practice guidelines
CSPs: cold‐stored platelets
CAR‐NK: chimeric antigen recepter natural killer
CAR‐T: chimeric antigen receptor T‐cell
DHQ: donor history questionnaire
ELGANs: extremely low‐gestational age neonates
ICTMG: International Collaboration for Transfusion Medicine Guidelines
PrEP: pre‐exposure prophylaxis
RCT: randomized controlled trial
SCD: sickle cell disease
TBI: traumatic brain injury
TDBT: transfusion‐dependent beta thalassemia
TM: transfusion medicine
TTIMS: Transfusion Transmissible Infections Monitoring System
TPE: therapeutic plasma exchange
tRAADP: targeted routine antenatal anti‐D prophylaxis
TTP: thrombotic thrombocytopenic purpura

## 1 INTRODUCTION

The Association for the Advancement of Blood and Biotherapies (AABB) Clinical Transfusion Medicine Committee (CTMC) annually reviews and summarizes critical developments in the fields of transfusion medicine (TM) and related disciplines for the AABB Board of Directors. Since 2018, this annual assessment has been published in *Transfusion* to serve as an educational resource for the global community. For the 2025 calendar year (CY2025) review, this collaborative effort was strengthened by participation of members from the AABB Biotherapies Leadership Committee (BLC). What follows is a synopsis of key advancements in TM articles published in calendar year 2025.

## 2 MATERIALS AND METHODS

Members of the CTMC and the BLC identified original research and significant articles published during CY2025. Selection was based on the perceived importance, originality, and potential impact on transfusion practice. Summary formats were narrative and evidence certainty was not systematically evaluated, although authors prioritized high‐quality trials and major clinical practice guidelines (CPGs). Each topic summary (created by 2–3 members) underwent an independent review and editing process by at least two separate committee members. The first and senior authors then compiled these summaries into the final manuscript, editing for length and thematic continuity.

## 3 BLOOD COMPONENT THERAPY

### 3.1 Key points

- The BORN trial reported that cord blood‐derived red blood cell (CB‐derived RBC) transfusion in extremely low‐gestational age neonates (ELGANs) may reduce severe retinopathy and bronchopulmonary dysplasia (BPD).
- The CRISP‐TBI trial found cold‐stored platelets (CSPs) were safe in traumatic brain injury (TBI).
- The CLIP‐II trial showed cryopreserved platelets were safe but less hemostatically effective than liquid‐stored platelets in cardiac surgery.

### 3.2 CB‐derived RBCs in neonates

In the BORN trial, ELGANs (24–27 weeks) across eight Italian hospitals were randomized to allogeneic RBC transfusions from either adult donors or CB to test the hypothesis that RBCs enriched with fetal hemoglobin may improve key neonatal outcomes.^1^ CB‐derived RBC units suspended in SAG‐M additive solution were prepared by Italian public cord blood banks and compliant with European regulations for RBC concentrates. In the intention‐to‐treat analysis (56 per arm), severe retinopathy rates were similar (28.6% vs. 25.0%), but 43% in the CB arm also received adult RBCs due to supply challenges (e.g., short shelf life, volume constraints, and processing/testing turnaround times). In the per‐protocol cohort (38 control, 17 CB), 34.2% and 26.3% of controls developed severe or treatment‐requiring retinopathy, whereas no neonate given exclusively CB‐derived RBCs developed these complications. Moderate/severe BPD rates also differed (63.2% vs. 29.4%). Hematocrit increments and safety profiles were comparable. Maintaining higher hemoglobin F percentages among transfused ELGANs may improve outcomes but needs further study.

### 3.3 Cold versus room temperature platelets in TBI

The CRISP‐TBI phase 2 open‐label randomized controlled trial (RCT) at a single US trauma center, compared CSPs versus room‐temperature stored platelets (RSPs) in patients with TBI.^2^ Patients received up to 2 apheresis CSPs stored up to 14 days versus standard RSPs. Six‐month functional outcomes did not differ, but CSPs were associated with fewer neurosurgical interventions (−14.4%, *p* = .03). Adverse events were similar, and CSP storage up to 14 days did not affect outcomes. Larger phase 3 trials are needed.

### 3.4 Cryopreserved versus liquid‐stored platelets in cardiac surgery

The CLIP‐II noninferiority RCT randomized 388 cardiac surgery patients to cryopreserved versus liquid‐stored platelets for surgical bleeding.^3^ Patients received up to 3 units of either cryopreserved platelets reconstituted in plasma or conventional liquid platelets in plasma, commencing intraoperatively or in the first 24 postoperative hours. Cryopreserved platelets did not meet the non‐inferiority threshold for 24‐h hemostatic effectiveness. Additional end points suggested diminished hemostatic efficacy, while adverse events were comparable.

## 4 INFECTIOUS DISEASES, DONOR SCREENING, AND COLLECTIONS

### 4.1 Key points

- Lenacapavir, HIV pre‐exposure prophylaxis (PrEP), was added to AABB Donor History Questionnaire (DHQ) v4.0 Medication Deferral List.
- HIV, HBV, and HCV incidence and window‐period residual risk all declined significantly from the 12‐month to 3‐month deferral period, with residual risk remaining under 1 per 2 million donations.
- Syphilis rates among US blood donors trended upward.
- Local transmission of Chagas Disease increased in the US

### 4.2 Lenacapavir and donor deferral

AABB DHQ v4.0 Medication Deferral List was updated July 2025 to include lenacapavir.^4^, ^5^ Lenacapavir, a long‐acting injectable HIV capsid inhibitor approved by the US FDA in 2025 for PrEP, provides up to 6 months of protection from a single injection.^4^, ^5^, ^6^ Because long‐acting PrEP medications can affect detection of early HIV infection, allogeneic blood donors who have taken lenacapavir should be deferred for 2 years from the last dose.^4^, ^5^

### 4.3 Transfusion‐transmitted infection trends

Analysis of the US Transfusion‐Transmissible Infections Monitoring System (TTIMS) found HIV incidence among first‐time US blood donors declined 2015–2023 despite shortening the deferral period for exposure risks from 12 to 3 months.^7^ Incidence decreased from 2.82 to 1.88 infections per 100,000 person‐years (PY) during the 12‐month versus 3‐month deferral periods, respectively (*p*< .05). A broader TTIMS analysis (2017–2023) examining HIV, HBV, and HCV across approximately 60% of the US blood supply found overall weighted incidence declined significantly (*p*< .01) for all three pathogens between the 12‐month and 3‐month deferral periods: HIV (2.22–1.03/100,000 PY), HBV (1.59–0.93), and HCV (0.53–0.16).^8^ Window‐period residual risk also declined for all three agents, with HIV decreasing from 0.45/million to 0.23/million donations. An 11‐year analysis of American Red Cross donations (2013–2023) found that syphilis infections in blood donors increased over the study period, paralleling US population trends.^9^

### 4.4 Local transmission of chagas disease within the US

Chagas disease, caused by *Trypanosoma cruzi*, is considered endemic to 21 countries in the Americas, excluding the US, but increasing evidence suggests local transmission within the US.^10^ Triatomine bugs are present in 32 states with Chagas infections documented in wildlife, domestic animals, and humans, including locally acquired cases in 8 states.^10^ From 2013 to 2023, Texas documented 50 probable or confirmed locally acquired cases.^10^ US blood donors are currently screened once by serology; the implications of increased local transmission for blood safety practices remain to be determined.

## 5 PATIENT BLOOD MANAGEMENT

### 5.1 Key points

- Updated Cochrane systematic review reaffirms restrictive RBC transfusion strategies do not increase 30‐day mortality in most clinical scenarios.
- Liberal postoperative RBC transfusion strategy in high cardiac risk veterans did not reduce death or major ischemic events.
- Liberal RBC transfusion strategy suggested for patients with acute myocardial infarction (AMI).
- New international platelet guidelines recommended restrictive transfusion strategies.

### 5.2 Meta‐analysis of RBC transfusion strategies

The updated Cochrane review on RBC transfusions compared restrictive versus liberal strategies across all RCTs. The number of eligible studies continues to expand: 61 adult (*n* = 27,639) and 8 pediatric trials (*n* = 2764); results found restrictive strategies reduced transfusion exposure by 42% without increasing 30‐day mortality in most clinical contexts.^11^ Several population exceptions were noted: patients with gastrointestinal bleeding had lower 30‐day mortality with restrictive transfusion (risk ratio [RR] 0.63), neurocritically ill patients had worse neurological outcomes at 6–12 months with restrictive strategies (RR 1.14), and the AMI population is discussed in a dedicated guideline section below.

### 5.3 Postoperative transfusion in patients with high cardiac risk

The TOP trial evaluated postoperative RBC transfusion strategies in 1424 veterans (97.8% male) with high baseline cardiac risk and postoperative anemia following major noncardiac surgery.^12^ A liberal transfusion strategy (Hb <10 g/dL) did not reduce the 90‐day risk of death or major ischemic events (RR 0.90; 95%CI 0.65–1.24) compared with a restrictive strategy (Hb <7 g/dL). However, non‐myocardial infarction cardiac complications (arrhythmias, heart failure, nonfatal arrest) were lower in the liberal group (5.9% vs. 9.9%; RR 0.59, 99%CI 0.36–0.98).

### 5.4 RBC transfusion guidelines in AMI

The AABB International CPG,^13^ the American College of Cardiology and American Heart Association,^14^ and the Canadian Cardiovascular Society^15^ suggested a liberal transfusion strategy for patients with AMI, noting that a restrictive strategy (Hb <7–8 g/dL) may result in increased mortality (AABB guideline absolute risk difference: 1.2%, 95%CI −0.6% to 2.6%). Conditional recommendations reflect the low certainty of evidence due to inconsistency across available trials and imprecision (somewhat wide confidence intervals in meta‐analysis).

### 5.5 Updated guidelines for platelet transfusions

The 2025 AABB and International Collaborative Transfusion Medicine Guidelines (ICTMG) international CPGs provided updated recommendations for platelet transfusion across diverse clinical settings, emphasizing restrictive transfusion strategies based on moderate‐to‐high certainty evidence for selected populations, and provide a platform for wider implementation.^16^ The guidelines include four strong recommendations and seven conditional recommendations, with platelet count thresholds ranging from no transfusion to <50 × 10^9^/L depending on clinical population.

## 6 HEMOSTASIS

### 6.1 Key points

- Four‐factor prothrombin complex concentrate (4F‐PCC) was superior to plasma for hemostatic response in cardiac surgery.
- In severe trauma, early factor concentrate resuscitation (fibrinogen concentrate plus 4F‐PCC) did not reduce transfusion amounts compared with frozen plasma.
- Updated International Society on Thrombosis and Hemostasis (ISTH) guidelines for disseminated intravascular coagulation (DIC) and thrombotic thrombocytopenic purpura (TTP).

### 6.2 PCC in cardiac surgery

The FARES‐II trial evaluated 4F‐PCC (initial dose ~25 IU/kg) versus plasma in adults with at least moderate bleeding and an international normalized ratio ≥1.5 after cardiopulmonary bypass.^17^ More patients in the 4F‐PCC group achieved a composite hemostatic response (77.9% vs. 60.4%; *p*< .001) with fewer serious adverse events and similar thromboembolic events and mortality. The findings align with contemporary cardiac surgery patient blood management guidelines^18^, ^19^; however, questions remain, regarding cost, optimal dosing, laboratory thresholds, and implementation.^20^, ^21^, ^22^

### 6.3 Factor concentrates versus plasma in trauma

The FiiRST‐2 trial, conducted across 6 Canadian trauma centers, randomized adults (*n* = 137) with massive hemorrhage protocol activation to receive either early factor concentrates (fibrinogen concentrate and 4F‐PCC) or plasma.^23^ There was no difference in allogeneic blood products transfused within 24 h, thromboembolic events, or mortality, suggesting factor concentrates are safe but not superior to plasma for initial trauma resuscitation.

### 6.4 Hemostasis‐related guidelines

ISTH updated the definition and scoring criteria for DIC to improve early identification.^24^ The ISTH focused update on TTP management is reviewed in the Apheresis section below.^25^

## 7 HEMOGLOBINOPATHIES

### 7.1 Key points

- Luspatercept improves hemoglobin levels in transfusion‐dependent beta‐thalassemia (TDBT).
- Mitapivat improves hemoglobin levels in patients with alpha‐ and beta‐thalassemia.
- Therapeutic anticoagulation improves outcomes in patients with sickle cell disease (SCD) and acute chest syndrome (ACS).

### 7.2 Luspatercept in beta‐thalassemia

The BELIEVE phase 3 RCT demonstrated that luspatercept, an erythroid maturation agent, provides long‐term efficacy and safety for treating anemia in TDBT.^26^ Those receiving luspatercept had at least a 33% reduction in transfusion burden compared to baseline, with sustained reductions in serum ferritin levels, reflecting both decreased iron load from transfusion and increased iron utilization for erythropoiesis. In non‐TDBT, the BEYOND study showed nearly 95% of patients receiving luspatercept achieved a hemoglobin increase ≥1.0 g/dL compared to 22.4% with placebo.^27^

### 7.3 Mitapivat in thalassemia and SCD

The oral pyruvate kinase activator, mitapivat, is FDA‐approved for adults with pyruvate kinase deficiency (2022) and thalassemia (2025), but under investigation for SCD. The ENERGIZE trial randomized 194 non‐transfusion‐dependent alpha‐ and beta‐thalassemia patients to mitapivat or placebo and demonstrated a hemoglobin response (≥1.0 g/dL increase from baseline) in 42% with mitapivat versus 2% with placebo (*p*< .0001).^28^ The related RISE UP trial in SCD showed hemoglobin response in 46% (mitapivat 50 mg) and 50% (mitapivat 100 mg) versus 4% with placebo (*p*≤ .0003).^29^

### 7.4 Therapeutic anticoagulation in patients with SCD and ACS

The TASC trial included 172 patients with SCD and ACS, demonstrating therapeutic tinzaparin for 7 days shortened ACS duration compared to prophylactic dosing (HR 0.71; *p* = .044) without major bleeding.^30^ Opioid use was lower with therapeutic anticoagulation.

## 8 PEDIATRICS

### 8.1 Key points

- Recent platelet transfusion CPG included recommendations for pediatric patients.
- Restrictive RBC transfusion strategies are increasingly used in extremely low birth weight (ELBW) infants.
- Management of hemolytic disease of the fetus and newborn (HDFN) varies internationally.
- A dynamic prediction model among preterm infants with severe thrombocytopenia predicted benefits and harms of platelet transfusion.

### 8.2 Updated platelet transfusion guidelines

All four strong recommendations in the 2025 AABB and ICTMG international CPG included pediatric patients.^16^ Restrictive transfusion strategies were recommended based on moderate‐to‐high certainty evidence for nonbleeding patients with hypoproliferative thrombocytopenia receiving chemotherapy or undergoing allogeneic stem cell transplant, preterm neonates without major bleeding, patients undergoing lumbar puncture, and patients with Dengue. One conditional recommendation for nonthrombocytopenic patients undergoing cardiovascular surgery included pediatric patients.

### 8.3 RBC and platelet transfusion trends in ELBW infants

An NHLBI REDS‐IV‐P study of transfusion thresholds in ELBW infants (2019–2023) found a decline in pre‐transfusion hemoglobin over time, but no change in pre‐transfusion platelet counts.^31^ The authors concluded the TOP^32^ and ETTNO^33^ trials appear to have influenced US transfusion practice, but findings from the PlaNeT‐2^34^ trial have not.

### 8.4 Postnatal variability in HDFN management

An international retrospective study of 1855 neonates with HDFN across 22 countries (2006–2021) demonstrated variability in postnatal management, including exchange transfusion frequency, intravenous immunoglobulin use, erythropoiesis‐stimulating agents, and RBC transfusion timing.^35^ Delivery after 37 weeks was associated with lower exchange transfusion rates and fewer adverse outcomes. The substantial practice variation underscores the need for standardized international guidelines.

### 8.5 Personalized platelet transfusion model in preterm neonates

A dynamic prediction model was developed to estimate the 3‐day risk of major bleeding or death in preterm infants with severe thrombocytopenia (<50 × 10^9^/L) if prophylactic platelet transfusion was given within 6 h versus withholding transfusion for 3 days.^36^ Derived from a multicenter cohort (*n* = 1042) and validated in a separate cohort (*n* = 637), the model demonstrated good performance and personalized risk predictions based on factors such as mechanical ventilation, necrotizing enterocolitis, and sepsis. Although outcomes were influenced by both platelet count and clinical status, transfusion benefit was most apparent at platelet counts <20 × 10^9^/L. The findings generally align closely with the AABB and ICTMG international CPG recommendation for preterm neonates, and use of individual patient factors for more personalized predictions is a priority for future research.^37^

## 9 IMMUNOHEMATOLOGY AND GENOMICS

### 9.1 Key points

- Novel approaches to mitigate anti‐CD47 interference
- Ethylenediamine tetraacetate/glycine acid (EGA) can dissociate cold reactive IgM antibodies from RBCs.
- Solid‐phase red cell adherence (SPRCA) demonstrated superior sensitivity for Kidd alloantibodies compared with gel column agglutination testing (GCAT).
- Targeted routine antenatal anti‐D prophylaxis (tRAADP) using fetal RHD screening reduced new D alloimmunization.
- Narrative summary of considerations when transfusing persons of East Asian ancestry.

### 9.2 Mitigating anti‐CD47 interference

Several strategies have been explored to mitigate anti‐CD47 interference with immunohematology testing, including modified anti‐IgG reagents, alloadsorption, and antigen masking with soluble CD47 or ALX148. Two more investigational approaches emerged in 2025: a recombinant SIRPα variant MagroEx and immunomagnetic beads (IMBs) coated with OVCAR3 lysate or recombinant CD47.^38^, ^39^ MagroEx neutralizes multiple anti‐CD47 agents without affecting detection of unexpected antibodies.^38^ The IMBs remove anti‐CD47 antibodies from plasma within 30 s while preserving other antibodies and maintain adsorption capacity for 3 weeks in preservation solution.^39^

### 9.3 EGA for dissociation of cold‐reactive IgM antibodies

EGA can effectively dissociate cold‐reactive IgM antibodies from RBCs to the same degree as dithiothreitol (DTT), while chloroquine diphosphate leaves significant residual IgM antibodies on RBCs.^40^ EGA treatment could be useful in cases of sensitization by high titer cold‐reactive IgM antibodies.

### 9.4 SPRCA superior for Kidd antibody detection

In a 7‐year retrospective study of 41 patients, SPRCA detected anti‐Kidd antibodies in 71% of cases missed or inconclusive by GCAT. Notably, 11 of these patients with discrepant results had recent transfusions, with 5 classified as delayed serologic transfusion reactions, 4 likely delayed hemolytic transfusion reactions (DHTRs), and 2 highly probable DHTRs.^41^ SPRCA's greater sensitivity may be particularly useful in recently transfused patients where Kidd antibodies are present at low titers that fall below the detection threshold of GCAT methods.

### 9.5 Targeted routine antenatal anti‐D prophylaxis (tRAADP)

A nationwide Danish registry‐based cohort study evaluating the D immunization rates before and after implementation of tRAADP found significantly reduced D immunization even with restricting prophylaxis to pregnancies confirmed‐positive with non‐invasive cell‐free fetal *RHD* genotypng.^42^ In 2010, Denmark became the first country to implement tRAADP, offering fetal *RHD* genotyping to all nonimmunized D negative pregnant women. The incidence of new D immunization decreased from 0.46% (95%CI 0.41–0.52; *p*< .001) in the pre‐tRAADP cohort (2004–2009) to 0.22% (95%CI 0.19–0.25) in the tRAADP cohort (2011–2018).

### 9.6 Transfusion considerations for persons of east Asian ancestry

A narrative review of data from South Korea, Japan, China, Hong Kong, and Taiwan highlights important blood group antigen and antibody differences in East Asian populations.^43^ Notably, the cis‐AB phenotype is relatively prevalent in Korea (0.04%) and can be mistyped by automated analyzers. Anti‐Mi^a^ and anti‐Di^a^ are more prevalent in East Asia, but commercial antibody screening panels from Europe/US typically do not include these antigens. The authors recommend using screening panels that include Mi^a^ or Di^a^‐positive cells for East Asian populations.

## 10 APHERESIS

### 10.1 Key points

- Therapeutic plasma exchange (TPE) remains widely used for neurological diseases, especially myasthenia gravis (MG).
- The ISTH update on TTP maintains strong recommendations for TPE plus corticosteroids for immune TTP (iTTP), with conditional recommendations for adding rituximab and caplacizumab. Recombinant ADAMTS13 is preferred over plasma for congenital TTP (cTTP) prophylaxis.
- The Endothelial Activation and Stress Index (EASIX) may predict treatment refractoriness and mortality in iTTP.
- In iTTP, adding caplacizumab to daily TPE and immunosuppression prevented refractoriness and exacerbations, but adoption of caplacizumab varies in the US.
- Insulin resistant, overweight, or obese plateletpheresis donors experienced substantial glucose and insulin changes.
- Specific formulas for obese men and women may prevent extreme over‐ or underestimation of total blood volume.

### 10.2 TPE utilization

Analysis of 20 years of World Apheresis Association registry data (2003–2023) and US national inpatient sample data set (2016–2021) note overall stable TPE utilization, with MG exacerbation remaining a particularly common indication.^44^, ^45^ However, a retrospective study examining 5 years (2019–2023) of TPE for neurological indications at a large academic medical center found declining use of TPE for MG coinciding with 2021 FDA approval of the neonatal Fc receptor inhibitor efgartigimod for MG.^46^

### 10.3 TTP guidelines

The 2025 focused update of the ISTH TTP guidelines reinforces TPE and immunosuppression as the standard of care in iTTP and maintains a conditional recommendation for adding rituximab and caplacizumab finding insufficient new evidence to change the strength or direction of the prior conditional recommendation.^25^ For cTTP in remission, the panel issued a strong recommendation favoring recombinant ADAMTS13 over plasma for prophylaxis, and a conditional recommendation favoring plasma over watchful waiting when recombinant ADAMTS13 is unavailable.

### 10.4 Real‐world study of caplacizumab

A 15‐center US study described 390 iTTP episodes (2017–2021), with 30‐day and 6‐month mortality rates of 3.7% and 7.4%, respectively.^47^ Most patients received TPE with plasma (median 6 procedures), and practice patterns varied substantially for replacement fluids, immunosuppression, and caplacizumab use. Even after FDA approval, caplacizumab was used in fewer than one quarter of eligible episodes; however, episodes treated with caplacizumab had zero reported 30‐day and 6‐month mortality in this dataset, compared with measurable mortality in those managed without the drug.

A large international real‐world study of caplacizumab in iTTP collected data from national TMA registries/centers for 1015 patients receiving caplacizumab added to daily TPE and immunosuppression (corticosteroids ± rituximab), compared with 510 historic controls who did not receive caplacizumab.^48^ Three‐month survival rate in the caplacizumab group was 98.5%, compared with 94% in controls. Patients receiving caplacizumab showed reduced refractoriness, exacerbations, and required fewer TPE sessions to achieve clinical response versus controls. The estimated number of patients needed to treat with caplacizumab to prevent one death was 22 and 3–4 to prevent any unfavorable outcome.

### 10.5 EASIX for predicting outcomes in iTTP

EASIX, calculated from creatinine, lactate dehydrogenase, and platelet count, has emerged as a promising endothelial injury surrogate in iTTP.^49^ In a multicenter cohort of 55 adults treated with contemporary regimens, higher baseline EASIX strongly predicted refractoriness and mortality, with day‐0 EASIX demonstrating good discriminative ability (AUC 0.81) and normalizing by day 7 in responders, well before ADAMTS13 recovery. EASIX remained low during ADAMTS13‐only relapses (low ADAMTS13 without clinical/hematologic features of microangiopathy) but spiked during clinical relapses, suggesting utility for monitoring, early identification of high‐risk patients, and potential integration into future treatment algorithms.

### 10.6 Apheresis and obesity

A prospective cohort study of 108 plateletpheresis donors found that donors who were insulin resistant, overweight, or obese experienced glucose and insulin changes up to threefold higher post‐donation than donors without these conditions (*p*< .001).^50^ While donors with normal weight or without insulin resistance returned to baseline glucose levels after a post‐donation meal, those with insulin resistance, overweight, or obesity did not recover within the 90‐min post‐meal observation period. The authors concluded that such donors may need assessment and education about these metabolic alterations during apheresis.

Modified formulas may provide better estimation of total blood volume in obese patients and thus could more accurately inform replacement fluid volumes for apheresis procedures.^51^, ^52^

## 11 BIOTHERAPIES

### 11.1 Key points

- Chimeric antigen receptor T‐cell (CAR‐T) and chimeric antigen receptor natural killer (CAR‐NK) therapies are rapidly expanding into autoimmune disease treatment.
- Regulatory T cells (Tregs) nearing FDA approval for graft versus host disease (GVHD) treatment.
- FDA approved the first cell‐based gene therapy for Wiskott‐Aldrich syndrome (WAS).
- FDA proposed a new regulatory pathway and removed risk evaluation and mitigation strategies (REMS) for CAR‐T.
- The UK enacted the first point‐of‐care manufacturing regulatory framework.

### 11.2 CAR‐T/CAR‐NK cell therapy for autoimmune disease

Since the first patient with systemic lupus erythematosus (SLE) was treated with CD19‐directed CAR‐T therapy in 2019, CAR‐based strategies have emerged as a promising therapeutic approach for autoimmune diseases. Currently, over 110 CAR‐T and approximately 10 CAR‐NK clinical trials in autoimmune diseases are active or recruiting on ClinicalTrials.gov.^53^ In a phase 1/2 basket trial using zorpocabtagene autoleucel (Zorpo‐cel/MB‐CART19.1), an autologous CD19 CAR‐T cell product, 22 of 24 patients achieved the efficacy endpoint: 9 of 10 patients with SLE achieved remission, 9 of 9 patients with systemic sclerosis showed no disease progression, and 4 of 5 patients with inflammatory myopathies achieved a moderate/major response. All patients remained off immunosuppressant medications over the 24‐week observation period.^54^

### 11.3 Clinical trials with Tregs

Tregs, formulated with hematopoietic stem cells and conventional T cells, have shown promise for the treatment of GVHD associated with allogeneic transplants. A phase 3 study [NCT0401368] with the product, now known as Orca‐T, an allogeneic T‐cell immunotherapy using donor Treg cells, showed 78% of patients achieved survival free of GVHD compared with 38.4% of patients treated with conventional allogeneic transplant.^55^ The Orca‐T biologics license application is under FDA review. Following the first approval of mesenchymal stromal cells for GVHD in December 2024, the approval of Tregs would be a major biotherapies milestone.

### 11.4 Gene therapy for WAS

Waskyra (etuvetidigene autotemcel) was approved by the FDA in December 2025 as the first cell‐based gene therapy for WAS.^56^ In patients with a *WAS* gene mutation, hematopoietic stem cells were genetically modified with a lentiviral vector encoding the *WAS* gene. Two open‐label, single‐arm, multinational clinical studies and an expanded access program with a total of 27 patients showed substantial clinical benefit. Treatment reduced the rate of severe infections by 93% in the 6–18 months post‐treatment and decreased moderate‐to‐severe bleeding episodes by 60% in the first 12 months post‐treatment, compared to the year prior to treatment.

### 11.5 Updates in regulatory landscape

The FDA articulated a new plausible mechanism pathway that allows approval of individualized therapies based on a credible biological mechanism and meaningful clinical benefit in a limited number of patients for whom RCTs are not feasible.^57^ The FDA also removed the risk evaluation and mitigation strategies (REMS) for autologous CAR‐T due to accumulated real‐world safety experience.^58^ Finally, the UK enacted the *Human Medicines (Amendment) (Modular Manufacture and Point of Care) Regulations 2025*, establishing the world's first dedicated regulatory framework enabling decentralized, point‐of‐care manufacturing of advanced therapies.^59^

## 12 HEALTH CARE DISPARITIES

### 12.1 Key points

- Persistent, multilevel barriers limit access to cellular therapies.
- Patients with SCD face barriers to equitable, comprehensive care.
- Increasing directed donation requests and proposed legislation may threaten blood supply stability.

### 12.2 CAR‐T immunotherapy

A scoping review (2017–2025) found that access to CAR‐T immunotherapy is disproportionately limited for racial/ethnic minorities.^60^ Barriers span multiple levels: patient (financial toxicity, low awareness, health literacy, mistrust, logistical challenges), provider (implicit bias due to insurance status and socioeconomic support, leading to delayed referrals despite clinical eligibility), institutional (centralization at academic centers), and policy (variable Medicaid coverage). These findings align with broader evidence that disparities in cellular therapies are driven by socioeconomic factors, healthcare infrastructure, and therapy‐related limitations, particularly in the US.^61^ Proposed solutions include standardized eligibility identification, decentralized allogeneic CAR‐T, and expanded support services, though evidence regarding effective interventions remains limited.

### 12.3 Structural inequities in SCD

US SCD mortality increased from 2008 to 2023, in contrast to declining cystic fibrosis outcomes.^62^ This disparity likely reflects underinvestment in SCD care, limited access to therapies like hydroxyurea, and challenges with high‐cost treatments. Uptake of gene therapy remains low due to costs (~$2 million per treatment) and infrastructure constraints. A *Social Science & Medicine* analysis argues that market priorities have eclipsed equity, and patient concerns about access and medical racism are often underrecognized.^63^ A Consensus Statement from the National Alliance of Sickle Cell Centers Consensus Recommendations on SCD Health Maintenance emphasizes comprehensive, equity‐focused care, including screening for social determinants of health, improved mental health access, and broader use of disease‐modifying therapies.^64^ A narrative review further frames barriers across global settings and emphasizes solutions such as community‐based care, policy reform, equitable research funding, newborn screening, telemedicine, and task‐sharing with trained health workers—particularly relevant in low‐resource settings.^65^ Overall, improving SCD outcomes requires both biomedical advances and coordinated efforts to address structural inequities and adequacy of health system infrastructures.

### 12.4 Blood supply

Increasing demands for socially motivated directed donation, particularly driven by fear and vaccine misinformation, and related non‐evidence‐based legislative action threaten to fragment the US blood supply, with particular risk to rural and underserved areas.^66^, ^67^, ^68^, ^69^ Directed donation carries infectious, immunologic, and financial risks to recipients, while imposing significant logistical, ethical, and operational burdens on blood centers and hospital blood banks that compromise inventory safety and stability.^67^, ^68^

## FUNDING INFORMATION

No funding was received for this research.

## CONFLICT OF INTEREST STATEMENT

E.P.C. reports personal fees from Plas Free‐Ltd. and Terumo BCT outside the submitted work. JWJ reports personal fees from Instrumentation Laboratory and Sanofi and research funding from Bayer AG and Diagnostica Stago. AKP reports personal fees from Octapharma. JA reports honoraria from Octapharma and Cerus. R.A.M. reports scientific advising for Cerus and Werfen; unrestricted education grants from Octapharma, Werfen, Haemonetics, and Hemosonics. No other authors have any conflicts of interest to disclose related to this manuscript submission.

## Data Availability

Data sharing not applicable to this article as no datasets were generated or analysed during the current study.
